# Combined Treatment with Low Concentrations of Decitabine and SAHA Causes Cell Death in Leukemic Cell Lines but Not in Normal Peripheral Blood Lymphocytes

**DOI:** 10.1155/2013/659254

**Published:** 2013-08-13

**Authors:** Barbora Brodská, Aleš Holoubek, Petra Otevřelová, Kateřina Kuželová

**Affiliations:** Institute of Hematology and Blood Transfusion, U Nemocnice 1, 12820 Prague 2, Czech Republic

## Abstract

Epigenetic therapy reverting aberrant acetylation or methylation offers the possibility to target preferentially tumor cells and to preserve normal cells. Combination epigenetic therapy may further improve the effect of individual drugs. We investigated combined action of demethylating agent decitabine and histone deacetylase inhibitor SAHA (Vorinostat) on different leukemic cell lines in comparison with peripheral blood lymphocytes. Large decrease of viability, as well as huge p21WAF1 induction, reactive oxygen species formation, and apoptotic features due to combined decitabine and SAHA action were detected in leukemic cell lines irrespective of their p53 status, while essentially no effect was observed in response to the combined drug action in normal peripheral blood lymphocytes of healthy donors. p53-dependent apoptotic pathway was demonstrated to participate in the wtp53 CML-T1 leukemic cell line response, while significant influence of reactive oxygen species on viability decrease has been detected in p53-null HL-60 cell line.

## 1. Introduction

Contemporary cancer therapy should fulfill requirements for targeted elimination of cancer cells simultaneously with minimal adverse effects. Methylation and acetylation of specific sites modulate chromatin structure and intensity of gene and protein expression levels, and subsequently, they regulate cellular pathways involved in cell cycle control and apoptosis. Epigenetic aberrations occur frequently in tumorigenesis [1–3], and genes silenced by abnormal methylation or acetylation are promising targets for cancer therapy approaches [4, 5].

Different DNA methyltransferases (DNMTs) ensure proper DNA methylation with different specificity for unmethylated or hemimethylated DNA. DNMT1 predominantly methylates hemimethylated CpG dinucleotides during the S phase maintaining the methylation pattern in the newly synthesized strand [6]. The role of DNMT3a and b is mainly in *de novo* DNA methylation [7]. DNMT1 expression is upregulated in certain malignant blood cells [8, 9], and DNMT3A mutation is the most frequent novel genomic variation in acute myeloid leukemias (AMLs) identified and characterized by parallel sequencing technologies [10]. It appears that the “maintenance DNA methylation” refers to the preservation of average levels of DNA methylation at certain regions, but not to an accurate copying of site-specific DNA methylation patterns [11]. DNMT1 was shown to bind p53 and to cooperate in antiapoptotic gene survivin promoter methylation in wt HCT116 cells but not in p53 null cells [12]. Methyltransferase inhibitors that competitively bind to the catalytic site of DNMT, such as 5-azacytidine (Vidaza), have been successfully used in clinical trials to treat myelodysplastic syndrome (MDS) [13]. Deoxyribonucleotide analog, 5-aza-2′-deoxycytidine (decitabine, Dacogen, DAC), significantly reduced global methylation compared with pretreatment baseline in cells of AML patients [14]. DAC-induced p53 and cell cycle arrest in G2/M phase have been reported in mouse embryonic fibroblasts (MEFs) with wtp53, while p53-null MEFs underwent apoptosis characterized by increase of cell fraction in subG1 phase and caspase 3 fragmentation [15]. 

Acetylation equilibrium is maintained by balanced ratio between histone acetylases (HAT) and histone deacetylases (HDACs) action. The activity of histones and many nonhistone proteins is regulated by the extent of acetylation of their lysine residues. Dysfunction of acetylation process is often associated with several diseases, especially cancer, and histone deacetylase inhibitors (HDACi) are used to epigenetically correct aberrant HDAC activity [16, 17]. Changes in gene transcription, direct induction of apoptosis, production of reactive oxygen species, and induction of cell cycle arrest have been proposed as the mechanisms of HDACi action [18]. Cell cycle arrest in G1 phase is widely documented as a consequence of HDACi-induced acetylation and transcription activation of p21WAF1 [19–21]. The acetylation status of p53 is extensively studied in connection with proapoptotic function of HDACi: loss of acetylation completely abolished p53-dependent growth arrest and apoptosis in HCT116 cells [22, 23].

Methylation and acetylation mechanisms are often interconnected, and they occur ubiquitously depending on one another. DNMT1 interacts with methyl-CpG binding proteins like MeCP2, which specifically recognizes fully methylated CpG sites, or with MBD3, both forming complexes with histone deacetylases HDAC1 and HDAC2 which in turn interact with DNMT1 [24, 25]. DNMT1 inhibition was also tested experimentally in cancer cells by alternate pathways using HDAC inhibitors that indirectly promote ubiquitin-dependent proteasomal degradation of DNMT1 [26]. Another mode of action of demethylating agents represents the release of HDACs from gene promoters resulting in transcription activation. This mechanism is responsible for cyclin-dependent kinase inhibitor p21WAF1 induction in AML-derived cells [27]. Therefore, combined action of drugs regulating methylation and acetylation is worth intensive investigation.

Combined treatment using two or more drugs is believed to bring more targeted effect in cancer therapy. Assessment of concurrent action of DNA methyltransferase inhibitor DAC and histone deacetylase inhibitor suberoylanilide hydroxamic acid (SAHA, Vorinostat) showed cooperation between these two drugs in suppressing colon carcinoma metastasis [28] or synergistic inhibition of Hey and SKOv3 cell growth by apoptosis and cell cycle arrest [25]. However, little is known about apoptotic proteins expression during combined treatment. Effect of such treatment on cells originating from nonsolid tumors has not been tested yet, although several clinical trials estimating combined effect of DAC and SAHA, administered concurrently or sequentially, on different forms of MDS, AML, or ALL are currently in progress (http://www.clinicaltrials.gov/). In our previous work, we observed augmented effect of DAC and SAHA, in combination, on cell proliferation, reactive oxygen species formation, and apoptosis in leukemic cell line CML-T1 [29, 30]. However, drug concentrations used in this research have been shown to dramatically affect also the viability of peripheral blood lymphocytes (PBL) of healthy donors. Therefore, action of low-drug concentrations inducing no or little adverse effect on normal cells has been studied in this work. The extent of apoptosis, cell cycle distribution, p21WAF1 expression changes, ROS formation, and apoptotic proteins expression were investigated in leukemic cell lines with different p53 status and in normal PBL. The mechanism of combined DAC and SAHA treatment with respect to the tumor suppressor p53 presence is discussed.

## 2. Materials and Methods

### 2.1. Cell Culture and Chemicals

Peripheral blood lymphocytes of healthy donors were isolated from buffy coats on Histopaque 1077 (Sigma-Aldrich) as described earlier [31]. Leukemia cell line CML-T1 and HL-60 (both from German Collection of Microorganisms and Cell Cultures, Braunschweig, Germany) were cultivated in RPMI 1640 (Biochrom AG, Germany) supplemented with 10% FCS, 37°C, and 5% CO_2_ atmosphere. Decitabine (Sigma-Aldrich) and SAHA (Cayman Chemical Company, Ann Arbor, MI, USA) were added from 1 mM stock solution to final concentration of 1 *μ*M for different times up to 48 h. Cytidine analogs azacytidine and decitabine are referred to be unstable in suspension [32]. Therefore, we investigated two modes of DAC action in cell lines: “one-shot" addition to final concentration of 1 *μ*M for 48 h, or sequential addition with fresh DAC added after 24 h. Moreover, two DAC concentrations were used in 24 h sequential mode: 0.5 *μ*M to reach the same total drug exposure as with one-shot added 1 *μ*M DAC or 1 *μ*M to maintain 1 *μ*M DAC concentration assuming that unstable decitabine degraded in the first 24 h. N-Acetyl-L-cysteine (NAC, Sigma-Aldrich) and caspase inhibitors z-VAD-fmk (Santa Cruz) and Q-VD-OPh (R&D Systems) were added to final concentration of 10 mM (NAC), 20 *μ*M (z-VAD-fmk), and 10 *μ*M (Q-VD-OPh), respectively.

### 2.2. Viability Test

The viability (metabolic activity) of cells preincubated in microplates with DAC and/or SAHA for 24 and 48 h was monitored using an MTT Kit I (Roche Diagnostics Corporation, Indianapolis, IN, USA). Following the procedure described earlier [31], the absorbance of reporter substrate was measured on an ELISA reader (MTX Lab Systems, Inc., Vienna, VA, USA).

### 2.3. Flow Cytometry

Distribution of cell cycle phases (monitored by propidium iodide, PI), generation of reactive oxygen species (ROS, observed by dichlorodihydrofluorescein diacetate, H_2_DCFDA), mitochondrial membrane potential (MMP, measured by MitoTracker Red, MTR), condensation of chromatin (DNA stained by Hoechst 33342), and the extent of apoptosis (defined by Annexin V-FITC positive/PI negative population of treated cells) were investigated by flow cytometry. All fluorescent probes were purchased from Life Technologies Corporation. All samples (50 000 events/sample) were analyzed on an LSRFortessa cell analyzer (BD Biosciences).

#### 2.3.1. Cell Cycle

Cells treated by DAC and/or SAHA for 24 or 48 h were harvested, washed twice with 2 mL of PBS, resuspended in 500 *μ*L of PBS, added into 4.5 mL of ice-cold 70% EtOH, and stored in −20°C. For the analysis, suspension was washed with PBS, resuspended in PI-staining solution (0.1% (v/v) Triton X-100; 100 *μ*g/mL RNaseA; 50 *μ*g/mL PI), and kept for 30 min in the dark.

#### 2.3.2. ROS Production and Mitochondrial Membrane Potential

Treated cells were harvested, washed with PBS, and suspended in 1 mL PBS. H_2_DCFDA, MTR, and Hoechst 33342 were added to final concentration of 10 *μ*M (H_2_DCFDA), 40 nM (MTR), and 2 *μ*M (Hoechst 33342) for 30 min in 5% CO_2_ at 37°C. 

#### 2.3.3. Apoptosis

Cells were harvested, washed with PBS, and resuspended in 100 *μ*L of Annexin Binding Buffer (ABB: 10 mM Hepes, pH 7.4, 140 mM NaCl, and 2.5 mM CaCl_2_) containing 5 *μ*L of Annexin V-FITC solution. After 15 min in the dark (RT), 400 *μ*L of ABB was added and suspension was incubated for another 5 min with 2 *μ*L of 250 *μ*g/mL PI stock solution.

### 2.4. RNA Isolation and qRT-PCR

RNA of total 5 × 10^6^ cells was isolated with RNeasy kit (Qiagen) according to manufacturers instructions and deposited in −80°C until use. mRNA quality and concentration were assessed on ND-1000 Nanodrop system, and qRT-PCR was performed on CFX96 real-time system (Bio-Rad) using SensiFAST SYBR No-ROX One-Step Kit (Bioline). Relative mRNA quantity and standard deviations were calculated with CFX Manager Software. *β*-Actin was used as reference gene. Primers for RT-PCR were designed with Primer-BLAST software (NCBI).

### 2.5. Immunoblotting

For immunoblotting, two ways of sample collection were used. The first method efficiently prevented unstable protein degradation and involved cell lysis directly to the Laemmli sample buffer. This procedure precluded quantification of protein concentration by Bradford reagent and it was used for samples from PBL, whose stability in usual lysis buffer was poor and its protein concentration was supposed to be stable, and for experiments with caspase inhibitors. For protein expression analysis from cell lines, standard lytic procedure was used: cells were lysed in Lytic Buffer (LB: 50 mM Tris. HCl pH 7.5, 1% NP-40, 5 mM EDTA, 150 mM NaCl, 1 mM DTT, 1 mM PMSF, 4 *μ*L/1 mL protease inhibitor cocktail, and 5 *μ*L/1 mL phosphatase inhibitor cocktail 2; both cocktails were from Sigma-Aldrich) for 30 min at 4°C and then centrifuged at 10.000 g/30 min/4°C. After protein concentration assay (Bradford) supernatants were mixed with 2xLaemmli sample buffer and boiled for 5 min. Five micrograms of total protein were then subjected to SDS-PAGE and transferred into nitrocellulose membrane (Hybond PVDF, Amersham). Rabbit polyclonal primary antibodies against Bcl-2, Bcl-x_L_, Bid, Mcl-1, p21, PARP, Puma, and survivin as well as mouse monoclonal anti-p53 and anti-caspase-3 (procaspase and active form) were from Santa Cruz Biotechnology. Mouse monoclonal anti-Bax originated from BioLegend and mouse monoclonal antibodies against caspases-7 and -8 (procaspase and active form), IAP, Smac, and XIAP were from BD Biosciences. Mouse monoclonal anti-*β*-Actin (Sigma-Aldrich) was used to detect *β*-Actin expression as control of equal loading. All primary antibodies, except anti-PARP (1 : 1,000) and anti-*β*-Actin (1 : 5,000), were used at dilution 1 : 500. Anti-rabbit and anti-mouse HRP-conjugated secondary antibodies were purchased from Thermo Scientific and used in concentrations 1 : 50,000–1 : 100,000. ECL Plus Western Blotting Detection System (Amersham) was used for chemiluminescence visualization and evaluation by G-box iChemi XT4 digital imaging device (Syngene Europe, Cambridge). Relative protein expression was evaluated by GeneTools Software and statistical analysis was performed in GraphPad software.

### 2.6. Fluorescence Microscopy

Cells in suspension were seeded on coverslip in humidified chamber for 15 min and then fixed with 4% paraformaldehyde (PFA) overnight. After 10 min of permeabilization by 0.5% Triton X-100 the cells were incubated for 1 h with mouse monoclonal anti-Bax (BioLegend) and rabbit polyclonal anti-COX (Cell Signaling Technology) primary antibodies (1 : 100) and for another 1 h with the mixture of secondary antibodies (Alexa Fluor488-conjugated anti-rabbit and Alexa Fluor647-conjugated anti-mouse, both from Life Technologies, 1 : 200) with Hoechst33342 (1 *μ*M, Life Technologies). After extensive washing by PBS-Tween, stained cells were mounted into ProLong mounting suspension and observed under confocal laser scanning microscope FluoView FV1000 (Olympus Corporation). Fluorescence images were processed by FluoView software FV10-ASW 3.1.

### 2.7. Statistical Analysis

 In diagrams, arithmetic means of at least four times repeated experiments were plotted with SEM error bars. Significance levels (*P* values of *t*-tests) were determined using InStat Software (GraphPad Software). A *P* value of 0.05 or lower was considered to be a statistically significant difference between groups compared.

## 3. Results

### 3.1. Cell Viability

**Figure 1:**
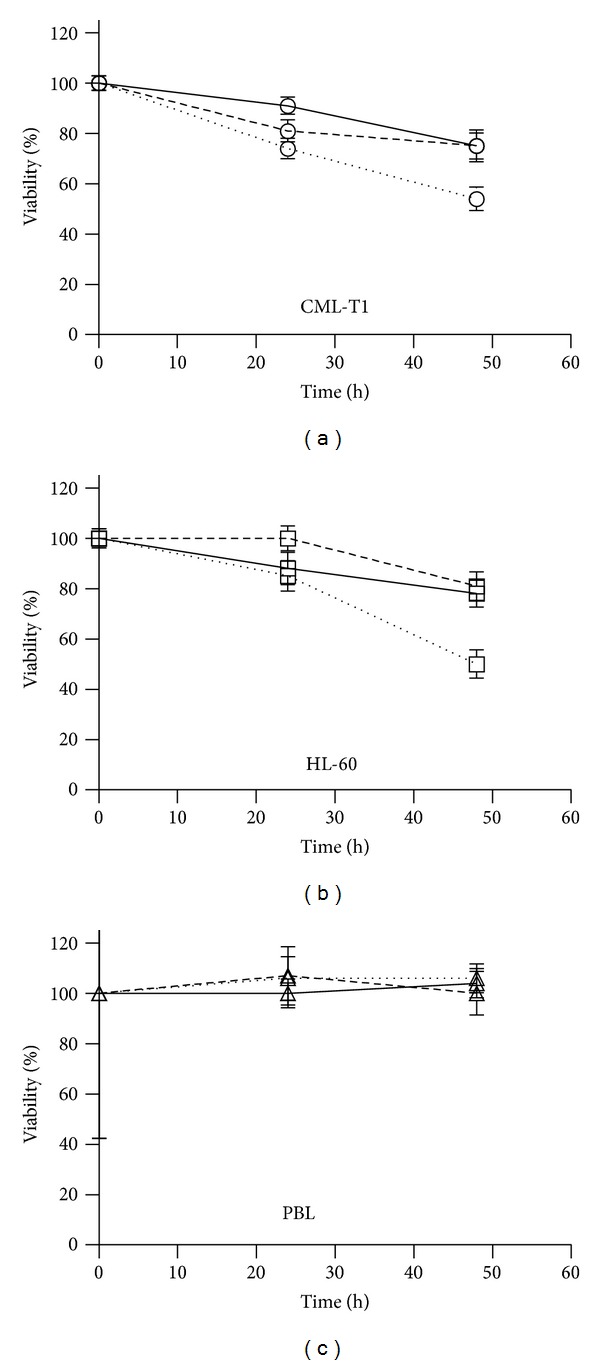
Effect of DAC and/or SAHA on cell viability during 48-hour treatment. Cells of leukemic cell lines CML-T1 (a, circles) and HL-60 (b, squares) or PBL (c, triangles) were treated by 1 *μ*M DAC (full line), 1 *μ*M SAHA (dash line), or 1 *μ*M DAC + 1 *μ*M SAHA (dotted line) in the 48 h time frame, and then their metabolic activity was measured by MTT test. Data are the average of at least five experiments and ±SEM are plotted as error bars.

The effect of 1 *μ*M DAC and/or 1 *μ*M SAHA on cell viability has been investigated during 48-hour treatment (Figure 1). In the 24 h time frame, DAC alone as well as SAHA caused slight viability decrease in CML-T1 cell line. Similar decrease after DAC treatment has been observed in p53-null HL-60 cells whereas no effect of SAHA was detected in HL-60 cell line. While the viability of lymphocytes was maintained at the control level even after 48 h of DAC + SAHA combination treatment, both cell lines, wtp53 CML-T1 and p53-null HL-60, showed significant viability decrease (*P* < 0.001).

### 3.2. Apoptotic Markers

**Figure 2:**
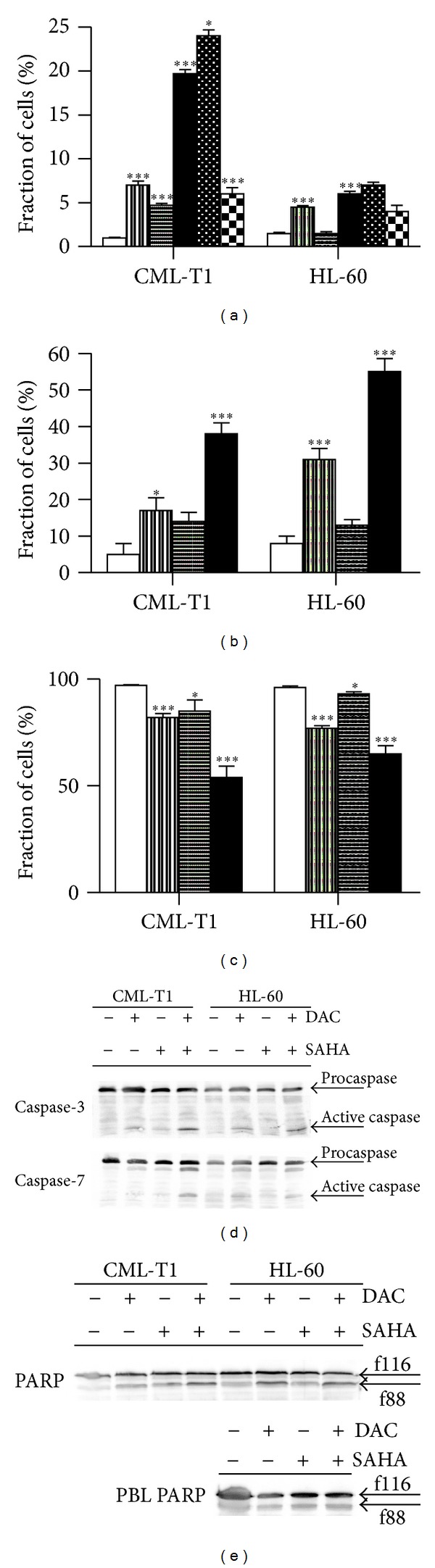
Apoptotic features induced by DAC and/or SAHA treatment: appropriate fraction of control cells (empty) or cells treated for 48 h by 1 *μ*M DAC (vertically shaded), 1 *μ*M SAHA (horizontally shaded), or DAC + SAHA combination (filled) measured by flow cytometry. (a) Annexin V-FITC/PI staining of nonfixed cells: Annexin V+/PI− fraction; effect of 10 *μ*M z-VAD-fmk (dotted) or 20 *μ*M Q-VD-Oph (checked). (b) PI staining of ethanol-fixed cells: fraction of cells in subG1 phase. (c) MitoTracker Red fluorescence: fraction of cells having polarized mitochondrial membrane. Error bars represent ±SEM of at least 3 measurements. Statistical significance degree of difference between treated samples and the corresponding control: *P* < 0.05 (*), *P* < 0.01 (**), and *P* < 0.001 (***). (d) Immunoblots showing caspases-3 and -7 activation after 48 h DAC and/or SAHA treatment. (e) Fragmentation of PARP in CML-T1 and HL-60 cell lines or PBL treated by DAC and/or SAHA for 48 h. Images are representatives of at least three experiments.

Annexin V+/PI− cell fraction, PARP fragmentation, executive caspases 3/7 activation, mitochondrial membrane depolarization, and fraction of cells in subG1 phase were analyzed to determine the extent of apoptosis induced by DAC and/or SAHA treatment (Figure 2).

**Figure 3:**
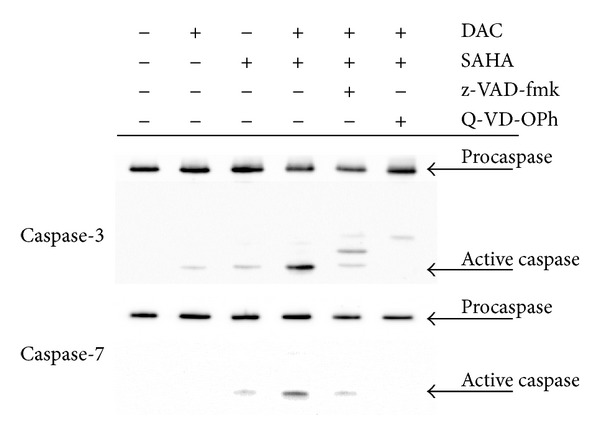
Inhibition of executive caspases cleavage by caspase inhibitors z-VAD-fmk (20 *μ*M) or Q-VD-OPh (10 *μ*M) in CML-T1 cells treated by 1 *μ*M DAC + 1 *μ*M SAHA for 48 h.

In all these apoptotic parameters, moderate changes caused by individual agents in CML-T1 or no apoptotic effect of SAHA on HL-60 cells in contrast to large apoptotic effect after 48 h of concurrent drugs action have been detected in both cell lines. Annexin V positivity was not influenced by 20 *μ*M caspase inhibitor z-VAD-fmk, but it was efficiently attenuated by 10 *μ*M Q-VD-OPh. Increase of Annexin V+/PI+ cell population induced by caspase inhibitors was observed in HL-60 cell line concurrently with Annexin V+/PI− fraction lowering (data not shown). This effect indicates damaging action of DAC and SAHA, which is not possible to reverse by caspase inhibition in HL-60 cell line. In CML-T1 cells, both used caspase inhibitors were able to effectively reduce caspase activation (Figure 3) causing 40% (z-VAD-fmk) and 60% (Q-VD-OPh) attenuation of active caspase-7 fragment, respectively, and 85% (z-VAD-fmk), respectively, complete (Q-VD-OPh) inhibition of active caspase-3 fragment. On the caspase-3 immunoblot, several intermediate products were visible in the presence of inhibitors. We did not detect any Annexin positivity, caspases activation, or subG1 peak in PBL (data not shown). Slight membrane depolarization and PARP fragmentation were observed after SAHA treatment of PBL, but these effects were not augmented by DAC addition.

### 3.3. Cell Cycle Arrest and p21 Induction

**Figure 4:**
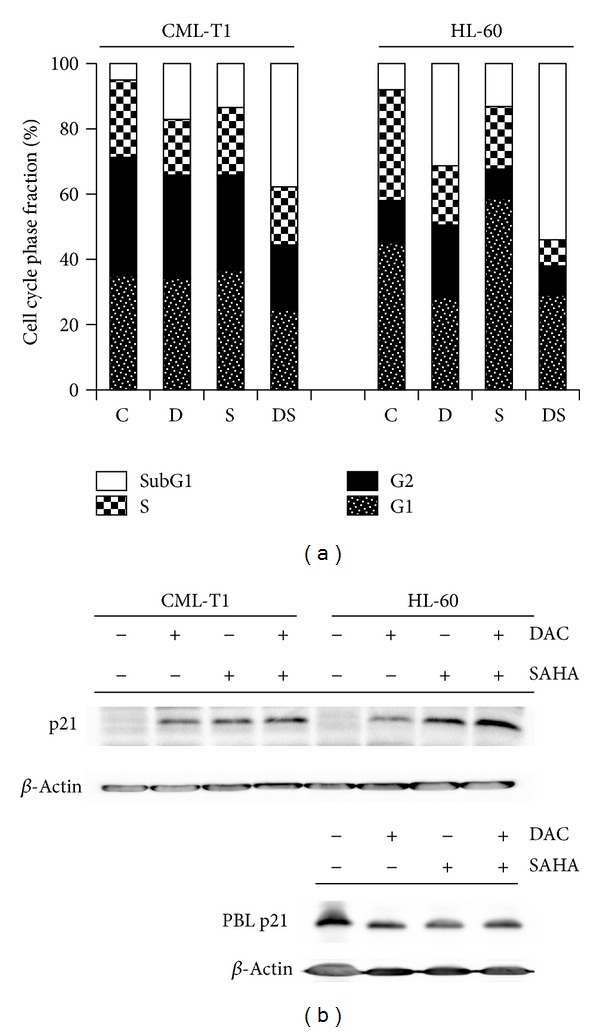
(a) Distribution of cell cycle phases monitored by PI staining of ethanol-fixed cells: untreated (C) or treated by 1 *μ*M DAC (D), 1 *μ*M SAHA (S), or DAC + SAHA combination (DS). Data are averaged from at least three experiments. (b) Expression of cyclin-dependent kinase inhibitor p21WAF1 after 48 h of 1 *μ*M DAC and/or 1 *μ*M SAHA treatment of leukemic cell lines (CML-T1, HL-60) or PBL. Images are representatives of at least five experiments.

Distribution of cell cycle phases was monitored by PI staining of ethanol-fixed cells during 48 h DAC and/or SAHA treatment (Figure 4(a)). Simultaneously, expression of cyclin-dependent kinase inhibitor p21WAF1, which is tightly connected with cell cycle progression from G1 phase, was examined (Figure 4(b)). Individual phases proportion was difficult to determine in CML-T1 cells due to their nearly tetraploid karyotype with diploid sideline. Nevertheless, p21WAF1 expression was substantially enhanced in all cases, that is, after DAC and/or SAHA treatment of CML-T1 cells. Decitabine increased G2-phase cell fraction, while SAHA caused an arrest in G1 phase in HL-60 cell line. The effect of combined treatment is mainly in large subG1 phase increase, but high proportion of nonapoptotic cells was found in G1 phase after DAC + SAHA treatment. Huge p21 induction was observed in both SAHA- and DAC + SAHA-treated samples, but increased p21WAF1 expression was detected also in DAC-treated HL-60 cells. No changes were observed in lymphocytes, which were almost complete in G0/G1 phase, the proportion of subG1 cells was up to 2% in PBL (data not shown) in all samples, and expression of p21WAF1 was maintained at the same level in all PBL tested samples. 

### 3.4. ROS Production

**Figure 5:**
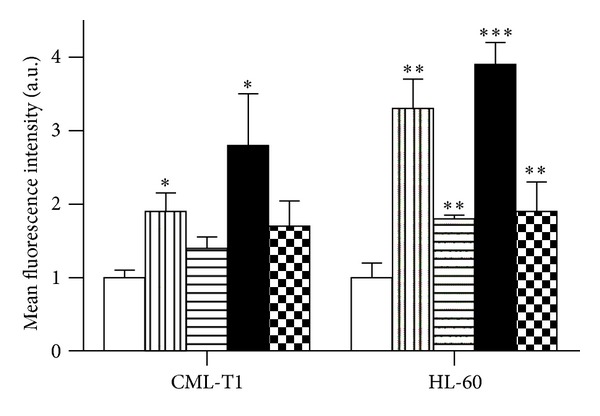
Production of reactive oxygen species. Control cells (empty) and cells treated for 48 h by 1 *μ*M DAC (vertically shaded), 1 *μ*M SAHA (horizontally shaded), or DAC + SAHA combination (filled) were stained by H_2_DCFDA, and the mean value of flow-cytometer-detected fluorescence intensity was analyzed. 10 mM NAC was used for DAC + SAHA-induced ROS generation reduction (checked). Error bars represent ±SEM of four measurements. Statistical significance degree of difference between treated samples and the corresponding control: *P* < 0.05 (*), *P* < 0.01 (**), and *P* < 0.001 (***).

Production of reactive oxygen species was monitored by H_2_DCFDA fluorescence intensity (Figure 5). Surprisingly, only slight ROS production was detected in any cells treated by 1 *μ*M SAHA alone. Decitabine alone or in combination caused H_2_DCFDA fluorescence distribution peak broadening but only moderate mean fluorescence value shift in CML-T1 cells. Contrary to this cell line, in HL-60 cells, DAC alone or in combination, induced a population with high H_2_DCFDA fluorescence intensity, and multicolor staining showed that this population is identical with the population of cells with depolarized mitochondrial membrane and condensed chromatin (more intensive Hoechst staining). Addition of 10 mM N-acetyl-L-cysteine (NAC) lowered the DAC-induced ROS production in both cell lines, but in CML-T1, NAC simultaneously induced mitochondrial membrane depolarization in comparison with control cells without NAC. Again, there were no considerable changes in ROS production in lymphocytes.

### 3.5. Mode of DAC Addition

**Figure 6:**
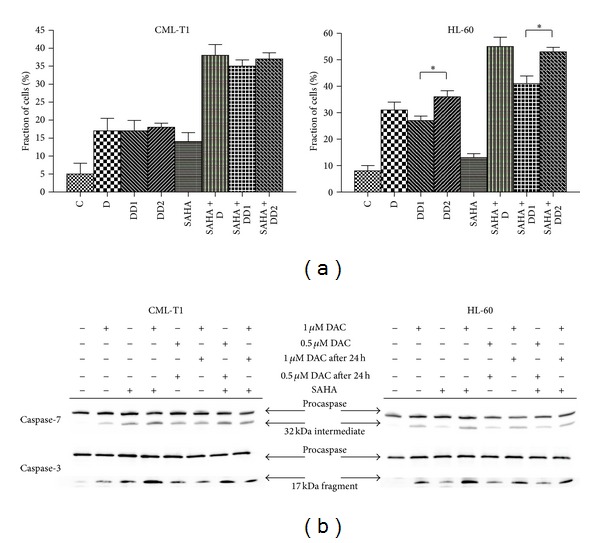
Apoptotic effects of one shot versus sequential DAC treatment. (a) Control cells (C) were treated by 1 *μ*M DAC for 48 h (D) or sequentially treated by two doses of 0.5 *μ*M DAC (DD1) or 1 *μ*M DAC (DD2) every 24 h in the presence or absence of 1 *μ*M SAHA. Treated cells were fixed in EtOH and stained by PI and the fraction of cells in subG1 phase of cell cycle was analyzed. Error bars represent ±SEM of four measurements. Statistical significance degree of difference: *P* < 0.05 (*). (b) Immunoblots showing executive caspases activation in samples are described in (a).

In CML, no substantial difference between the samples with 0.5 *μ*M and 1 *μ*M DAC was observed, and the effect was comparable also for sequential DAC addition versus one-shot mode. Effect of sequential DAC addition into HL60 cell suspension, either with or without SAHA, was strongly influenced by DAC concentration. 0.5 *μ*M repeated DAC caused significantly lower effect (*P* < 0.05) in all parameters (cell viability decrease, mitochondrial membrane depolarization, fraction of cells in subG1 phase, PARP fragmentation, executive caspases activation, ROS generation, and p21 expression) than 1 *μ*M DAC. On the other hand, 1 *μ*M DAC induced nearly identical effect independently of the mode of addition in HL60 cells. Fraction of cells in subG1 phase and executive caspases activation are presented in Figure 6 as representative results of these phenomena.

### 3.6. mRNA Expression

**Figure 7:**
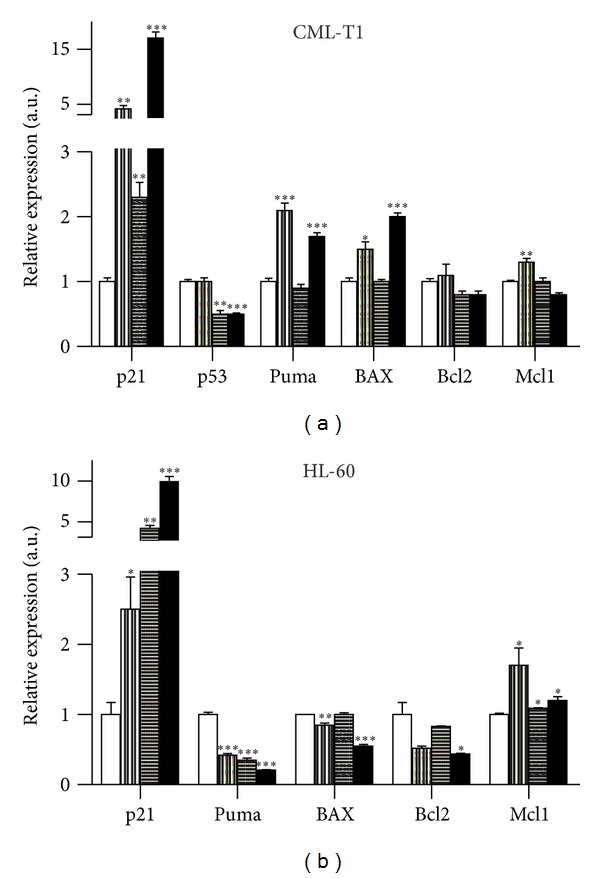
Transcription levels of cyclin-dependent kinase inhibitor p21WAF1, tumor suppressor gene TP53, and several apoptosis-related genes in leukemic cell lines treated by 1 *μ*M DAC and/or 1 *μ*M SAHA monitored by qRT-PCR. Error bars represent ±SEM of three measurements. Statistical significance degree of difference between treated samples and the corresponding control: *P* < 0.05 (*), *P* < 0.01 (**), and *P* < 0.001 (***).

The transcription levels of cyclin-dependent kinase inhibitor p21WAF1, tumor suppressor gene TP53, and several apoptosis-related genes were investigated by qRT-PCR (Figure 7). Massive p21WAF1 gene induction has been observed in both individual agents and combination-treated cell lines. Expression of TP53 was not influenced by DAC, it was lowered by SAHA or combination in CML-T1 and, of course, not detectable in p53-null HL-60 cells. Genes for proapoptotic proteins Bax and Puma were induced by DAC in CML-T1 but even decreased in HL-60, where Bcl-2 gene was also attenuated after DAC + SAHA treatment. Surprisingly, Mcl-1 induction was observed, namely, in HL-60 cells, indicating activation of some antiapoptotic mechanism. No statistically significant changes were detected in transcription levels of the antiapoptotic protein Bcl-xL. 

### 3.7. Protein Expression

**Figure 8:**
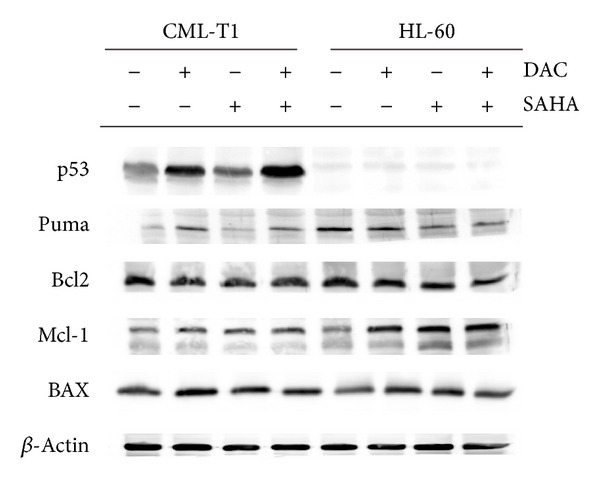
Expression of tumor suppressor p53 and proteins related to apoptosis in control cells and after 48 h DAC, SAHA, or DAC + SAHA treatment of leukemic cell lines monitored by immunoblotting. Images are representatives of at least four experiments with similar results.

Expression of tumor suppressor p53 and proteins related to apoptosis was studied by immunoblot (Figure 8). p53 expression was induced by DAC in CML-T1 cells independently on concurrent SAHA presence. Together with p53 induction, increase of p53-upregulated mediator of apoptosis Puma expression was observed. No p53 expression change has been documented in lymphocytes and, of course, in p53-null HL-60 cell line, where Puma level diminution was simultaneously detected. Contrarily, slight decrease of antiapoptotic protein Bcl-2 level was detected after combined treatment of HL-60 but not in CML-T1. Induction of Mcl-1 observed on transcription level was detected also in protein expression. Only minimal increase of protein expression after treatment was detected for proapoptotic Bax. We did not detect any change in expression of several other apoptosis-related proteins: Bcl-xL, BIM, IAP, XIAP, Smac, and survivin.

### 3.8. Fluorescence Microscopy

**Figure 9:**
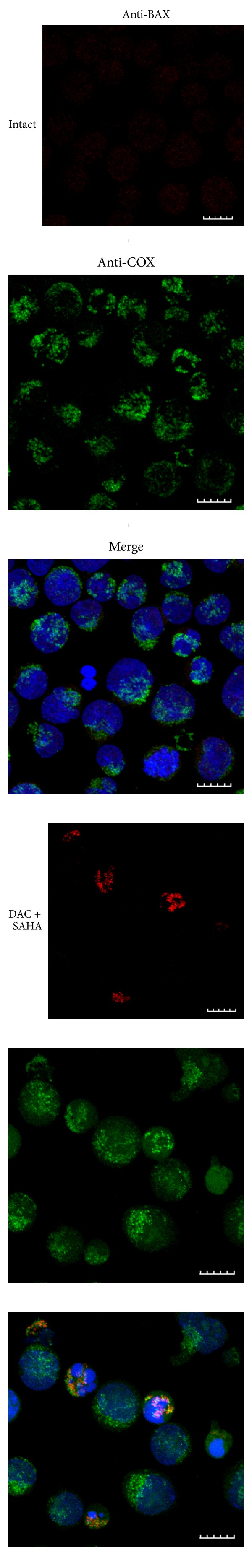
Intact cells CML-T1 and cells treated by DAC + SAHA combination for 48 h stained with anti-Bax (red, Alexa Fluor647) and mitochondrial anti-COX (green, Alexa Fluor488) antibodies. Nuclei are visualised by Hoechst 33342. Scale bar represents 10 *μ*m.

Activation of proapoptotic Bax during its translocation from cytoplasm into mitochondrial membrane is widely described as one step of the intrinsic apoptotic pathway. Enhanced Bax gene expression in CML-T1 cells was not reflected by substantial increase of Bax protein level, but fluorescence microscopy revealed redistribution of Bax from whole cellular volume into mitochondria in apoptotic cells (Figure 9). On the other hand, Bax localization in HL-60 was nearly mitochondrial even in intact cells, and concentration of fluorescence signal into mitochondria in apoptotic cells after DAC and/or SAHA treatment was not accompanied by significant fluorescence intensity change (data not shown).

## 4. Discussion

The effectiveness of chemotherapy drugs may be improved by administering them in combination (combination chemotherapy). Combination drug therapies can target multiple pathologic processes, and lower drug doses allow for minimizing the adverse side effects. Moreover, *de novo* and acquired resistance arises with molecularly targeted drugs and cytotoxic chemotherapy, limiting their utility, and rational combinatorial targeted therapy can help to overcome this problem [33].

Many combination therapies are used to treat metastatic breast cancer [34]. Several inhibitor combinations effective for melanomas with activating RAS or BRAF mutations were recently discovered [35]. Combination therapy of *α*-galactosylceramide and 5-fluorouracil showed antitumor effect in mice and suggests a new therapy mode against metastatic liver cancer [36]. More pronounced effect of rapamycin and docetaxel combination has been manifested against nonsmall-cell lung cancer [37]. However, several combination therapies revealed excessive adverse effect and bad tolerability [38] or were not improving effect of single drugs [39]. In this paper, combination of drugs affecting two fundamental epigenetic processes, methylation and acetylation, has been investigated on cell lines originating from leukemia patients. The effect on wtp53 CML-T1 line and p53-null HL-60 line was compared with the effect on PBL of healthy donors. The viability assay manifested no effect of individual DAC or SAHA treatment on PBL, which remained unaffected even when combined treatment is applied. By contrast, marked viability drop after 48 h of combined DAC + SAHA treatment has been detected in both cell lines tested, irrespective of their p53 status, surpassing the individual drug effects. Several apoptotic features were further analyzed to determine the extent of apoptosis induced by individual drugs and their combination on the cell lines with different p53 status. While annexin V positivity, PARP fragmentation, and caspases-3 and -7 activation correlated with viability drop observed in CML-T1 cells, all these changes had only moderate extent in HL-60 cells. Caspase inhibitors effectively blocked caspases-3/7 cleavage, but even increasing apoptotic cells fraction in Annexin V-FITC staining was observed using 20 *μ*M z-VAD-fmk inhibitor. It is likely that z-VAD-fmk-induced inhibition of executive caspases fragmentation is not reflected by entire recovery of cellular processes and cells are directed to cell death despite blocked caspases. Indeed, unspecific damaging effect of this inhibitor was recently observed in renal injury [40]. On the other hand, comparable extent of mitochondrial membrane depolarization was detected in both cell lines tested. Most of the parameters tested remained unchanged after DAC and/or SAHA treatment of PBL. The only effect of SAHA alone or in combination with DAC on PBL was slight mitochondrial membrane depolarization and PARP fragmentation, which was not accompanied by viability drop or executive caspases activation. As epigenetic action of DAC targets S-phase fraction of cells, prevalent appearance of lymphocytes in G0/G1 phase of cell cycle is the possible reason of high lymphocyte resistance to low DAC concentration exposure.

Cell cycle analysis brought information about significant proportion of cells, namely, of HL-60 line, in subG1 phase when treated by DAC and SAHA. While interpretation of cell cycle distribution of CML-T1 cells was complicated by partial tetraploidy of CML-T1 cell line, cell cycle arrest in G1 after SAHA treatment and G2 phase arrest after DAC action was evident in HL-60 line. However, both of these individual drug effects were overlaid by marked increase of cells in subG1 after combined drug action. Cell cycle arrest is usually in tight connection with cyclin-dependent kinase (cdk) inhibitors; therefore, we tested the gene and protein levels of p21WAF1, which are known to inhibit several cdks, mainly cdk2. In CML-T1 cells, both gene and protein levels were significantly enhanced after individual drug action and also after combination treatment. Large p21WAF1 expression increase was detected also in HL-60 treated by SAHA or by DAC + SAHA, while only moderate enhancement was observed in DAC-treated HL-60. This is in agreement with findings of other authors describing direct effect of HDACi on p21WAF1 promoter acetylation and its expression induction [41, 42] and p53-induced p21WAF1 activation of wtp53 cells after DAC treatment [43]. Induction of p21WAF1 by DAC, even in p53-null HL-60 cells, suggests another mechanism of p21WAF1 expression stimulation. One of such possible mechanisms is reactive oxygen species (ROS) formation induced by DAC treatment occurring especially in p53-null cells [44, 45]. Recently, direct relation between DAC-induced DNMT1 depletion and p21WAF1 induction was observed in drug-resistant breast cancer cells (MCF-7/ADR) [46]. 

Both DAC and DAC + SAHA treatments induced ROS generation in both cell lines tested, but this effect was more pronounced in HL-60 cell line. Moreover, N-acetyl-L-cysteine (NAC) protected p53-null HL-60 cells more effectively than wtp53 cell line CML-T1. The main mechanism of DAC action on HL-60 cells is therefore probably ROS production causing enhanced p21 expression and moderate level of apoptosis. Relatively low extent of apoptotic changes in comparison with high-treatment cytotoxicity and persistence of PI-positive cell fraction in the presence of caspase inhibitors indicate that another type of cell death is induced in parallel with the apoptosis in HL-60 cells. ROS formation, p21WAF1 gene and protein induction, cell cycle arrest, and also DAC-induced increase of cell volume imply possible role of senescence [47–49], the mechanism of DAC action which has been observed in prostate cancer cells [50] or in malignant pleural mesothelioma [51]. Induction of senescence has been detected also in SAHA-treated colon cancer cells with nonfunctioning p21WAF1 or p53 genes [52]. 

Although DAC is reported to have short half-life in suspension, we did not detect any substantial difference between the samples with 1 *μ*M DAC, that is, fresh 1 *μ*M DAC added into treated cell suspension after 24 h of action had no significant effect. Contrarily, sequential 0.5 *μ*M DAC induced significantly lower effect than the 1 *μ*M DAC in HL60, regardless of SAHA presence. No difference was detected in CML which indicates that although 1 *μ*M DAC induces comparable effect in both of tested cell lines, CML, but not HL60 line, is equally sensitive even to lower drug concentration. The effects on apoptotic parameters are probably initiated in the first 24 h of DAC action, before its depletion, and therefore no effect of additional drug dose has been observed. 

Analysis of apoptotic gene and protein expression changes revealed p53-dependent apoptotic way of cell death in CML-T1 cells. Attenuated degradation by proteasome should be the mechanism of DAC-induced boost of p53 protein expression, because the increase in protein level is not preceded by TP53 gene induction. Both the gene and protein expressions of p53-inducible protein Puma were enhanced in concert with p53 stabilization. Although we detected increase of Bax mRNA in CML-T1, only minimal change has been observed on active Bax protein level. However, fluorescence microscopy revealed high accumulation of active Bax in mitochondria of apoptotic cells. Bax participation on apoptosis is mediated by its relocalization into the mitochondrial membrane [53]. Our results show that this relocalization is not necessarily accompanied by large change of overall Bax expression. 

## 5. Conclusion

Combined treatment with epigenetic drugs was tested in cell lines originating from nonsolid tumors (leukemia) with respect to their p53 status. Participation of proteins related to apoptosis was investigated during individual as well as combined drug treatment and multiple apoptotic features were monitored. In summary, combination of decitabine and SAHA markedly decreased the viability of leukemia cell lines. While in wtp53 cell line apoptosis was induced also by individual agents alone and their effect in combination was additive, p53-null cells seemed to be sensitized by SAHA, which in itself was not toxic for HL-60, for enhanced reaction in response to DAC presence. Tumor suppressor p53 and its downstream targets Puma and Bax were identified to play the main role in apoptosis in wtp53-possessing CML-T1 cell line, while ROS formation was found to affect cell death in p53-null HL-60 cell line. The absence of negative effect on normal peripheral blood lymphocytes nominates this combination of anticancer drugs to the group of possible therapeutic tools that are profitable for further investigation.
